# A real-world analysis of the impact of X-linked myotubular myopathy on caregivers in the United States

**DOI:** 10.1186/s13023-025-03583-w

**Published:** 2025-05-12

**Authors:** Tina Duong, Tmirah Haselkorn, Beckley Miller, Julie Coats, Ivar Jensen, Erin Ward, Marie Wood, Robert J. Graham, Laurent Servais

**Affiliations:** 1 Stanford University, 213 Quarry Road, Palo Alto, CA 94041 USA; 2 Astellas Gene Therapies, San Francisco, CA USA; 3 PRECISIONheor, Boston, MA USA; 4 MTM-CNM Family Connection, Inc, Methuen, MA USA; 5 Division of Critical Care Medicine, Boston Children’s Hospital, Harvard Medical School, Boston, MA USA; 6 MDUK Oxford Neuromuscular Centre & NIHR Oxford Biomedical Research Centre, University of Oxford, Oxford, UK; 7 Division of Child Neurology, Centre de Références Des Maladies Neuromusculaires, Department of Pediatrics, University Hospital Liège & University of Liège, Liège, Belgium

**Keywords:** XLMTM, Myotubular myopathy, Congenital myopathy, Rare disease, Quality of life, Economic impact, Caregiver, Real-world data

## Abstract

**Background:**

X-linked myotubular myopathy (XLMTM) is a rare, life-threatening congenital myopathy with multisystem involvement, which often includes the need for invasive ventilator support, gastrostomy tube feeding, and wheelchair use in approximately 80% of patients. The direct and indirect financial impact of extensive supportive care, as reported by caregivers of individuals with XLMTM, and the health-related quality of life (HRQoL) of caregivers has not been previously described. Here, we use a survey co-designed by patient advocates to provide objective information on the physical and financial challenges of caregiving for individuals with XLMTM.

**Methods:**

A real-world web-based survey was conducted in the United States between November 19, 2019, and January 23, 2020. The survey was developed in association with patient advocacy leaders from the XLMTM community, who were also caregivers of individuals with XLMTM. The survey included the EuroQol 5-dimension 5-level HRQoL instrument and visual analog scale, and a cost (direct and indirect medical costs) and healthcare resource questionnaire. The survey was shared among the XLMTM community by patient advocacy organizations. Caregivers who completed the survey and met the eligibility criteria were included. Descriptive statistics were conducted using Microsoft Excel.

**Results:**

Twenty-two caregiver respondents agreed to participate. All respondents completed the cost and health resource survey. Productivity loss varied between participants over the prior 12 months. Durable medical equipment expenses comprised most of the direct medical out-of-pocket costs. Non-medical expenditures (e.g. home and vehicle modifications) were higher than direct medical out-of-pocket costs. Twelve of the 22 respondents completed the HRQoL survey. The HRQoL domains most impacted were usual activities, anxiety/depression, and pain/discomfort.

**Conclusions:**

Findings from this real-world survey of caregivers for individuals with XLMTM describe the caregiver experience, as well as the multifaceted impact of the disease on caregiver productivity loss, out-of-pocket expenses, and HRQoL. XLMTM comes with financial constraints and substantial impacts on caregivers’ physical and mental health. Understanding these gaps is crucial to support the caregivers who provide care for this medically fragile population.

**Supplementary Information:**

The online version contains supplementary material available at 10.1186/s13023-025-03583-w.

## Introduction

X-linked myotubular myopathy (XLMTM) is a rare disease caused by mutations in the *MTM1* (myotubularin 1) gene, resulting in the absence or dysfunction of myotubularin, a ubiquitously expressed enzyme that is required for normal development and function of skeletal muscle cells [1–3]. The estimated global incidence of XLMTM is approximately one in 40,000–50,000 newborn males, with approximately 80% experiencing severe symptoms such as profound muscle weakness, hypotonia, and the inability to establish spontaneous respiration [4–7]. Life expectancy for individuals with XLMTM is poor. Approximately 50% die by 18 months of age, and the median lifespan is 29 months [4, 6, 8]. Individuals with XLMTM require lifelong care from birth, with a high proportion (85–90%) requiring respiratory support at birth and two-thirds requiring permanent mechanical ventilation [4, 8].

There are currently no approved disease-modifying therapies for XLMTM. Symptom management includes long-term supportive care such as ventilatory support (delivered invasively via a tracheostomy or non-invasively via a mouthpiece or mask), gastrostomy tube feeding, and musculoskeletal management [9, 10]. Given the complexity of XLMTM, supportive care is often administered and managed by a multidisciplinary team which may include: neurologists; pulmonologists; orthopedists; physical, occupational, and speech therapists; assistive technology specialists; respiratory specialists; and gastrointestinal specialists [5, 11, 12]. In recent years there has been an increased awareness of the potential for liver-related symptoms of XLMTM. As such, the inclusion of hepatologists in XLMTM multidisciplinary care planning will be important for the effective management of this complex disorder [13].

Individuals with XLMTM have high rates of healthcare utilization, hospitalization, and surgical intervention, with the caregivers in one study reporting losing a collective average of one day of work a month per surveyed family [14]. However, the real-world consequences of an XLMTM diagnosis on caregivers of affected individuals, in terms of both health-related quality of life (HRQoL) and financial well‑being, have not been comprehensively described. This novel US-based survey aimed to describe the physical, emotional, and financial impact of XLMTM on caregivers, including costs (such as direct medical, direct non-medical, and out-of-pocket expenses), healthcare resource use, and association with HRQoL.

## Methods

### Study design

A quantitative web-based survey was developed for caregivers of individuals with XLMTM. Patient advocacy leaders, who were also caregivers of individuals with XLMTM, reviewed and co-designed the survey questions in partnership with the survey team. The survey was conducted in the United States between November 19, 2019, and January 23, 2020. The objectives were to evaluate the economic impact of XLMTM, both work-related and financial, for caregivers of affected individuals, and to evaluate the caregiver HRQoL.

The survey design and content were reviewed and approved by the Advarra Institutional Review Board to be used for research purposes. The survey was conducted according to the principles expressed in the Declaration of Helsinki. Written informed consent was obtained from all participants.

### Participants

Participants were included in the study if they were a caregiver or parent (≥ 18 years of age) of a male individual diagnosed with XLMTM who was not enrolled in the ASPIRO clinical trial (ClinicalTrials.gov identifier NCT03199469), a resident of the United States, and able and willing to provide written informed consent. Only one caregiver response per individual with XLMTM was permitted; if an individual with XLMTM had more than one caregiver, only one was permitted to respond to the survey. Henceforth, the term “participant” will be used to reference the caregiver in this report and “individuals with XLMTM” will be used for those for whom the caregiver is providing care.

Patient advocacy organizations in the United States, including the Joshua Frase Foundation (Florida; https://www.joshuafrase.org/), MTM-CNM (Myotubular-Centronuclear Myopathy) Family Connection (Massachusetts; http://www.mtm-cnm.org/), and Where There’s a Will There’s a Cure (Illinois; https://www.will-cure.org/) disseminated information about the survey to the XLMTM community via emails, newsletters, websites, social media, and word of mouth. Interested caregivers were then directed to the online survey hosted on the Qualtrics platform (Supplementary Material 1). Upon accessing the survey website, participants were provided with information on the survey objectives and eligibility criteria before being asked to provide their consent for all responses to be used for analysis and subsequent publication.

### Survey content

The cost and healthcare resource questionnaire was comprised of nine sections and 36 questions. Briefly, participants were asked to provide information on demographics for themselves and for the individual with XLMTM, including age, which was captured in years. Mean caregiving years was assumed to equal the age of the individuals with XLMTM. In cases where the individual with XLMTM’s age was given as > 18 years and the number of caregiving years was between 18 and 25, the age was noted as the corresponding caregiving years. When the number of caregiving years was categorized as > 25, the age of the individual with XLMTM was assumed to be > 25 years, unless otherwise noted in the open-ended qualitative responses. Individuals with XLMTM aged > 25 years were grouped together. Participants were also asked questions to help describe the physical characteristics and impact of disease on the individual with XLMTM. Other questions focused on cost (direct medical, non-medical, out‑of‑pocket, and indirect work-related impact) and healthcare resource use.

Caregiver HRQoL was assessed using the EuroQol 5-dimension 5-level (EQ‑5D-5L) HRQoL instrument and visual analog scale (EuroQol-VAS) (Supplementary Material 2) [15]. The EQ-5D-5L questionnaire covers five dimensions: mobility, self‑care, usual activities, pain/discomfort, and anxiety/depression. Each dimension has five response levels ranging from one (no problems) to five (unable to/extreme problems), and the respondent selects one answer. Caregiver self-assessed overall health status was recorded by selecting a number on the vertical EuroQol-VAS, between zero and 100, where zero represents “the worst health you can imagine” and 100 represents “the best health you can imagine.”

All questions were optional, and no pretesting was performed. Omitted responses were censored from the analyses. For omitted cost values where utilization was selected (e.g. wheelchair use = “yes”, but cost was left blank) the cost was assumed to be $0. As the Qualtrics survey platform was unable to integrate the EQ-5D-5L tool into the survey, respondents were required to download and complete the instrument, and independently return the completed tool by one of three methods. The first was to either upload or send to the study group contact provided; the second was to upload directly to Qualtrics; and the third was to request contact to complete by telephone. The full questionnaire can be found in Supplementary Material 2, with additional information on data collection in *Supplementary Methods.*

Participant data for analysis were anonymized. No identifying information was shared with the sponsor. All respondent data were backed up by Qualtrics using automatic propagation across servers (immediate upon collection) and a daily complete off-site encrypted backup.

### Statistical analysis

The number and percentage of hours of both paid and unpaid caregiving needs were calculated, summarized, and combined to determine the total hours of caregiving needs. Annual unpaid caregiver productivity loss was calculated as: estimated productivity lost/year = (median hourly pay rate [$29.86] in 2023) × (total employment hours reduction/week/family) × 52 weeks/year + (median hourly pay rate [$29.86] in 2023) × (days missed related to caring for an individual with XLMTM) × 8 work hours/day [16].

Scores on each of the EQ-5D-5L dimensions were converted into an index score representing a von Neumann-Morgenstern utility value for current health state [15]. Health states were converted into weighted health state indices (utility) using the EQ-5D-5L preference weights elicited from general population samples as a reference [17]. Weighted health state index (utility) scores range from zero to one, where zero denotes quality of life as death and one denotes the value of full health. For this study, US population weights were applied in the conversion to an EQ-5D-5L index (utility) score [18]. The decrease in the quality of life (i.e. utility) of caregivers was measured as disutility [19]. The disutility score for each caregiver was calculated as: disutility score = EQ-5D-5L index score – US population–weighted EQ-5D-5L index score at the corresponding age range, with lower scores being associated with lower HRQoL.

Both non-response errors and responses that were outside the scope of the answer set were described for each measure, and generated data were distinguished in the presentation of results. Descriptive statistics were conducted using Microsoft Excel.

## Results

### Demographics and characteristics of caregivers

Twenty-two caregiver participants (median age [interquartile range, IQR]: 44.5 years [11.0 years]) responded and consented to participate; only 12 (55%) participants completed the EQ-5D-5L instrument. Characteristics of caregivers are presented in Table 1. Participant-level data are presented in Supplementary Table S1.

**Table 1:** Characteristics of caregivers and individuals with XLMTM

Characteristic	Caregivers (N = 22)*
Median age (IQR), years	44.5 (11.0)
Sex, female, n (%)	17 (76)
Highest level of education, n (%)	
*Completed high school/GED*	2 (9)
*Bachelor's degree*	10 (46)
*Master's degree*	6 (27)
*Other degrees*	4 (18)
Median caregiving years (IQR)	17 (16.75)
Median number of additional unpaid caregiver (IQR)	2 (1.75)
Median proportion of unpaid caregiving hours (IQR)	46 (29.11)

Individuals with XLMTM	
Characteristic	Individuals with XLMTM (N = 22)
Number alive at the time of the survey, n (%)	19 (86)
Median age (IQR), years	16 (12.5)
Invasive ventilation, n	14
BIPAP, n	6
Supportive O_2_, n	6
SIMV, n	6
Pressure support, n	7
IPPV, n	1

^*^Number of patients/caregivers with characteristic provided, unless otherwise specified

BIPAP, bilevel positive airway pressure, GED, General Equivalency Diploma; IPPV, intermittent positive-pressure ventilation; IQR, interquartile range; SIMV, synchronized intermittent mandatory ventilation, XLMTM, X-linked myotubular myopathy

The majority of caregivers who completed the survey were female (76%); nearly half (46%) had earned a bachelor’s degree and nearly one-third a master’s degree (27%). The number of paid (i.e. externally hired) and unpaid caregiving hours (including travel time, medical visits, and night-time care) was 0−168 h/week and 0–226 h/week, respectively. The median percentage of unpaid caregiving hours was 46.2% (IQR: 29.1%) of all the caregiving hours needed.

### Demographics and characteristics of individuals with XLMTM

Characteristics of individuals with XLMTM are presented in Table 1, and participant‑level data are presented in Supplementary Table S1. Nineteen of the 22 (86%) individuals with XLMTM (median age [IQR]: 16 years [12.5]) were alive at the time of the survey. Approximately 64% (n = 14) of individuals with XLMTM were receiving invasive ventilation. Caregiver-reported characterization of motor, feeding, and speaking milestones was completed for all individuals with XLMTM and described below.

#### Motor milestones

Fourteen (64%) participants reported that the individual with XLMTM had no loss in motor milestones from the highest achieved milestones (Supplementary Table S2A). Seven (32%) participants had a reported decline of ≥ 1 milestone, two of whom had more significant declines of two major milestones: one individual with XLMTM who once walked unaided but lost the ability to sit without support or roll, and another individual with XLMTM who lost the ability to sit or roll. One response was excluded due to a discrepancy in the responses, wherein the best lifetime milestone was lower than the reported current milestone.

#### Feeding milestones

Twenty (91%) of the individuals with XLMTM had no change in their feeding status from best to current (Supplementary Table S2B). Nineteen (86%) individuals with XLMTM required a nasogastric or gastric tube throughout their life. A few individuals were able to eat by mouth by either independent feeding (n = 1), use of an assistive device (n = 1) or by parents/caregiver (n = 1). One response was excluded due to the best lifetime milestone being lower than the reported current milestone. This participant was different from the one excluded from the motor milestone description.

#### Speaking and communication milestones

At the highest achieved milestones, only one (5%) individual with XLMTM “spoke with no difficulties”, 10 (45%) “spoke with some difficulties”, three (14%) “communicated with assistive communication devices”, three (14%) “communicated with sign language”, two (9%) were “unable to speak or communicate”, and three (14%) used “other” to describe the speaking level (Supplementary Table S2C). Three (14%) reported a net loss between best and current or last milestone for the individuals with XLMTM: one communicated with sign language and lost ability to speak/communicate before passing away; two individuals lost ability to speak clearly but were able to communicate using a speaking valve with a tracheostomy.

### Caregiver productivity, costs, and healthcare resource utilization

#### Caregiver productivity

**Fig. 1:**
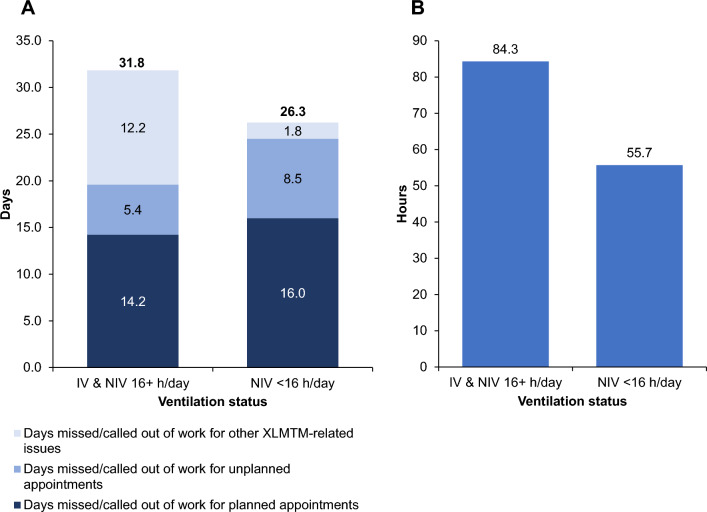
Caregiver productivity loss by ventilation status for: (**A**) mean days missed/called out of work in the last 12 months; and (**B**) mean total weekly hours spent caring for the individual with XLMTM. h, hour, IV, invasive ventilation, NIV, non-invasive ventilation, XLMTM, X-linked myotubular myopathy

Participant responses on impact to income, employment, and productivity are presented in Table 2. Nineteen participants were estimated to have a median loss of productivity of $33,324 per year (IQR $46,462). Participant-level data are presented in Supplementary Table S3. Caregiver productivity loss by ventilation status was higher for caregivers whose individuals with XLMTM required ventilation for ≥ 16 h/day compared with caregivers whose individuals with XLMTM required ventilation for < 16 h/day (mean days missed/called out of work in last 12 months: 31.8 vs. 26.3 for ≥ 16 h/day compared to < 16 h/day, respectively) and is presented in Fig. 1.

**Table 2:** Caregiver productivity and financial costs

Caregiver Productivity	Caregivers (N = 22)
Family lost one full income – refuse promotion or change career, n (%)	
* Gave up paid employment completely*	4 (18)
* Refused promotions/changed career goals*	4 (18)
* Both: Gave up paid employment completely and refused promotions/changed career goals*	4 (18)
* Neither: Did not give up employment or change career goals*	10 (45)
Median (IQR) caregiver employment reduction, hours/week/family	24.0 (25.0)
Median (IQR) caregiver missed workdays for planned appt (past 12 months)	7.5 (13.5)
Median (IQR) caregiver missed workdays for unplanned appointment (past 12 months)	2.5 (7.0)
Median (IQR) caregiver missed workdays XLMTM-related issue (past 12 months)	4.0 (5.0)
Median (IQR) productivity loss/year, $	$33,324 ($46,462)
**Median (IQR) annual direct medical out-of-pocket costs (N = 22)**	**Cost ($)**
Wheelchair	$0 ($175)
Assistive technology	$81 ($407)
Disposable medical equipment	$339 ($1,831)
Ventilation	$0 ($0)
Medical procedures	$0 ($0)
Medical therapy	$0 ($119)
Total direct medical	$1,392 ($2,160)
**Median (IQR) annual indirect medical out-of-pocket costs (N = 22)**	**Cost ($)**
Home modifications	$528 ($1,776)
Vehicle modifications	$2,450 ($5,877)
Total indirect medical	$4,188 ($6,370)
**Median (IQR) annual expenses (N = 22)**	**Cost ($)**
Additional vacation expenses	$0 ($875)
Caregiver training	$0 ($0)
School transportation	$0 ($0)
HCP visits with travel	$500 ($775)
Specialist school costs	$0 ($0)
Total	$950 ($1,675)
**Median (IQR) annual financial assistance (N = 22)**	**Cost ($)**
SSDI assistance	$29 ($1,454)
Non-SSDI assistance	$435 ($5,556)
Total financial assistance	$1,812 ($5,407)

Estimated productivity loss/year = (median hourly wage [$29.86] in 2023) × (total employment hours reduction/week/family) × 52 + (median hourly pay rate [$29.86] in 2023) × (days missed related to XLMTM) × 8

HCP, healthcare provider; IQR, interquartile range; SSDI, Social Security Disability Insurance, XLMTM, X-linked myotubular myopathy

#### Costs

Median annual direct and indirect medical out-of-pocket costs across all participants were $2,442 (IQR $2,160) and $6,287 (IQR $6,370), respectively (Table 2). Median annual expenses were $950 (IQR $1,675). Participants received a median of $1,812 (IQR $5,407) in annual financial assistance. Participant-level data are presented in Supplementary Table S3.

#### Healthcare resource utilization

The median number of healthcare providers or specialists’ visits per month for individuals with XLMTM was 1.5 (IQR 2.75). Sixteen (73%) of the individuals with XLMTM spent < 3 h/week traveling to healthcare providers or specialists (eight of 22 spent < 1 h/week, four of 22 spent 1–2 h/week, and four of 22 spent 2–3 h/week). Of the six individuals with XLMTM who spent > 3 h/week traveling to healthcare providers or specialists, two spent 3–4 h/week, two spent 4–5 h/week, and two spent 5–6 h/week. Seventeen of the 22 individuals with XLMTM spent < 1 week in hospital in the 12 months prior to completion of the survey. Among the five of 22 individuals with XLMTM who spent > 1 week in hospital during the last 12 months, two were infants (≤ 1 year) who spent 4–5 months in hospital. The other three individuals with XLMTM were deceased, and spent 2 weeks, 2 weeks, and 3 months in hospital during the last 12 months of their life.

### Caregiver health-related quality of life

**Fig. 2:**
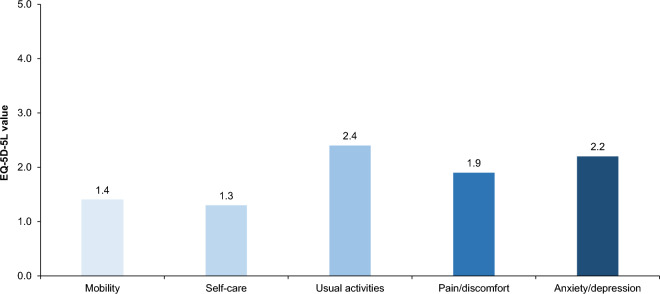
Mean EQ-5D-5L index values for respondents (N = 12) who completed the survey. Outlier values were excluded. Each dimension has five response levels ranging from one (no problems) to five (unable to/extreme problems). Abbreviation: EQ-5D-5L, EuroQol 5-dimension 5-level

**Fig. 3:**
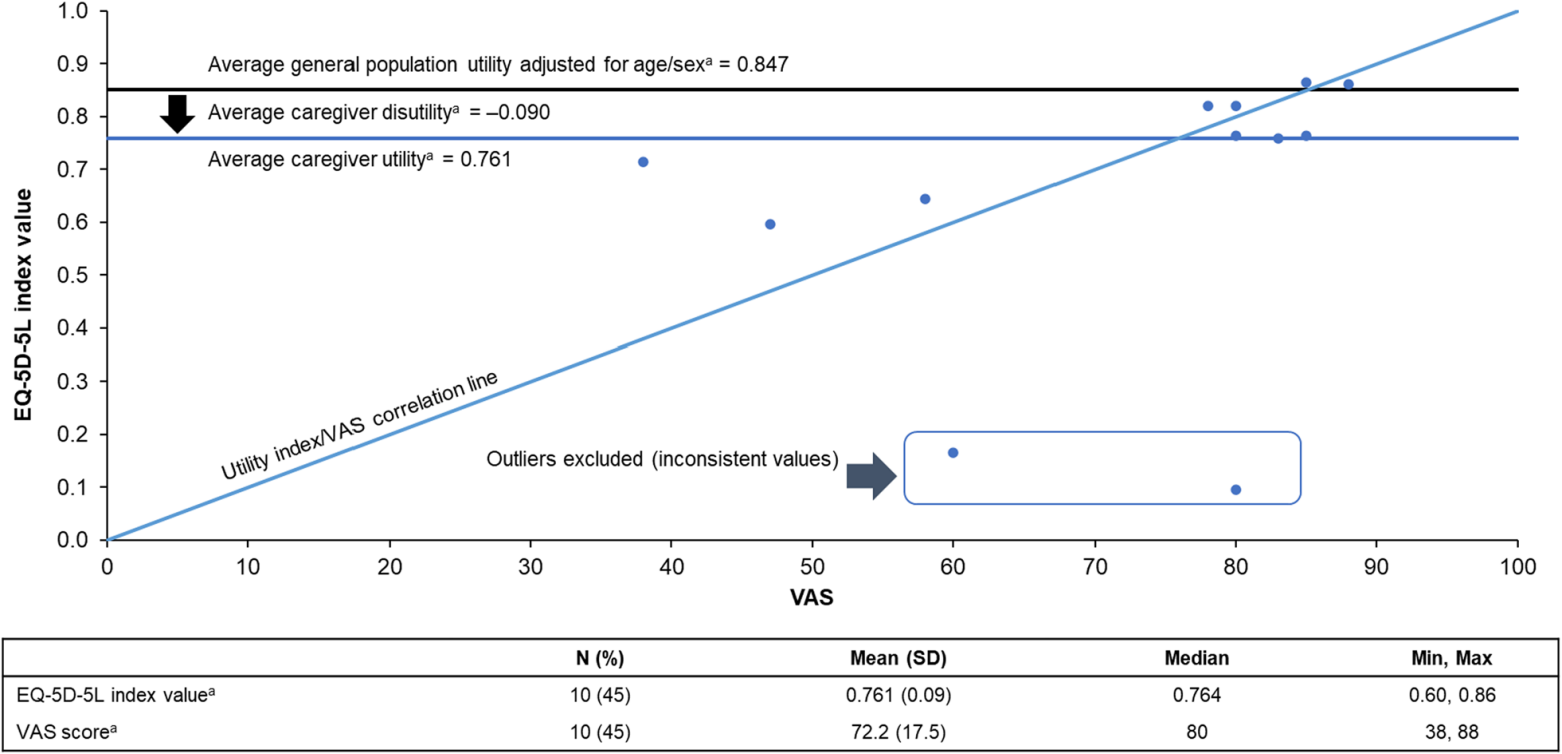
Comparison of VAS and EQ-5D-5L index values for respondents (N = 12) who completed the EQ-5D-5L questionnaire. ^a^Outlier values reported near 0 EQ-5D scores and incongruent VAS scores and thus were excluded when calculating means. EQ-5D-5L, EuroQol 5-dimension 5-level; SD, standard deviation; VAS, visual analog scale

Twelve of 22 (55%) participants completed the EQ-5D-5L questionnaire to assess caregiver HRQoL. The percentage of caregivers with living individuals with XLMTM in the population that completed the HRQoL survey (11 of 12 responses [92%]) was comparable to the overall population of caregivers with individuals with XLMTM who were alive at the time of the survey (19 of 22 [86%]). The mean scores of all 12 respondents in five dimensions (individual index scores and VAS) are shown in Fig. 2. The mean (n = 10; outlier values excluded) EQ-5D-5L index value (utility) of the caregivers was 0.761 (median 0.764; IQR 0.20; Fig. 3), and the mean caregiver disutility was − 0.090. The mean VAS was 72.2 (median 80; IQR 24.0). Participant-level data are presented in Supplementary Table S4.

### Open-ended comments

Twelve (55%) participants provided a response to the open-ended questions at the end of the survey (Table 3). The responses are summarized below, grouped into those related to financial and HRQoL impact:

**Table 3:** Summary of open-ended comments on financial and HRQoL aspects of the survey

	Open-ended comments from the participants
Financial aspects	Endemic costs/difficulty in quantifying (three families)
Difficulty in quantifying Endemic costs	1. It was difficult to estimate the costs of travel including gas to get to appointments and time it takes to load and unload their child and find suitable parking
Endemic costs	2. It was difficult to estimate the additional cost of having their child at home even through adulthood with care-givers coming in and out of the home and the increase in utilities, toilet paper, electricity, laundry, etc.
Difficulty in quantifying Changing over lifetime	
Endemic costs	3. Annual expenses seemed very arbitrary since the current situation reported only the last 12 months whereas these expenses may have been higher in other life phases of the individual with XLMTM’s
Endemic costs	4. The survey did not capture the need to move homes (at a financial loss) due to needing to support their child’s medical needs (hundreds of thousands of dollars)

Financial aspects	Job loss and job retention (two families)
Difficulty in quantifying Changing over lifetime	5. The caregiver was unable to work for first 18 months of their son’s life that caused productivity loss of hundreds of thousands of dollars
Difficulty in quantifying Changing over lifetime	6. The caregiver was unable to work for the first 20 months of the life of their son with XLMTM

Financial aspects	**Endemic costs/difficulty in quantifying (three families)**
Difficulty in job retention Nursing needs	7. The caregiver changed jobs multiple times due to the excessively flexible schedule needed to accommodate the nursing needs of the individual with XLMTM
Nursing needs Overall financial cost	8. The caregiver spent personal funds to provide nursing when not covered by insurance; the total expenses over 30 years were up to $1.5 million
Nursing needs Impact on work Sleep deprivation	9. The nursing shortage required the parents (unpaid caregivers) to take shifts staying up, and then try to function for work and/or taking care of the individual with XLMTM on the next day for 5 years

Financial aspects	Insurance premiums (two families)
Insurance premiums Overall financial costs	10. The largest portion of out-of-pocket expenses (besides vehicle modification) was $5,000 medical deductible for copays/co-insurance for doctor visits, specialty medications not covered by insurance; the insurance deductible was quickly met each year throughout the life of the child
Insurance premiums Self-insured employment Multiple insurance policies Changing lifetime needs	11. The survey did not consider medical insurance premiums; primary insurance from an employee-owned self-insured company was paid from the company’s bottom line, which affected the stock value, dividends, bonuses, and salaries of the company The caregiver had a second private insurance plan for 2 years to cover costs that were not covered by the primary or state insurance plans, adding thousands of dollars in premiums

Financial aspects	Overall financial (two families)
Overall financial costs	12. Both parents worked full-time jobs to afford everything their son with XLMTM needs “to have the best life possible. It is very expensive to care for him”
Overall financial costs	13. Caring for a disabled child has severely impacted their financial future

HRQoL	Physical, social, and emotional impact not captured
Physical impact Feelings of helplessness Social impact Stress/anxiety Depression Fear	14. The caregiver reported physical impacts, including tendinitis in wrist (needing immobilization for 5 months), torn rotator cuff, herniated/bulging disk issues, high blood pressure, weight gain, and feelings of helplessness to gain control over own health issues The caregiver also reported that they had no real social ties outside of their family unit – friends gone, some family too; feelings of loneliness and isolation adding to the stress and anxiety and being in fight-or-flight every day
Physical impact carrier of XLMTM Feelings of helplessness Nursing needs Fear	15. The caregiver reported the impact of wear and tear on the caretaker’s body for caring, which was already compromised as being a carrier of XLMTM along with other serious conditions The caregiver reported the helpless feeling of losing the ability to care for the individual with XLMTM due to own physical problems The caregiver reported the feeling of being scared, fearing that if their child were to be placed in a group home then they would soon die due to the amount of tracheostomy and oral suctioning they require
Sleep deprivation Stress/anxiety Fear Coping versus living with	16. The EQ-5D-5L did not seem to capture any detail regarding health impact and quality of life of caregivers, such as lack of sleep, level of intensity of caregiving/responsibilities, emotional stress of continuously facing possibility of child’s death, and providing life-saving interventions on a daily basis The caregiver stated that their ability to cope and a strong support system did not mean that the impacts were not significant, even if their “overall health” score was relatively high
Stress/anxiety PTSD	17. Caring for a disabled child has left them with severe PTSD which currently impacts their daily life
Stress/anxiety Social impact Single parent	18. The caregiver reported concerns over the sustainability of their emotional well‑being. They have given up all church involvement and nearly all social involvement The caregiver stated that their life was consumed with caring for the son with XLMTM and this was a daunting amount of work to do as a single parent
Feelings of helplessness Fear	19. The caregiver was not able to lift child any longer and was unable to afford a ceiling lift

EQ-5D-5L, EuroQol 5-dimension 5-level; HRQoL, health-related quality of life; PTSD, post‑traumatic stress disorder; XLMTM, X-linked myotubular myopathy

#### Responses on financial impact

Eight (36%) participants had concerns that the survey did not capture the full financial impact on their family, including endemic costs that were difficult to estimate and quantify, such as the increase in household utilities related to round-the-clock nursing care, dynamic expenses that vary according to stage of life (e.g. the loss of a job or the need to stop working which may be a temporary requirement), nursing need (e.g. the need to pay for nursing care not covered by insurance), and financial implications of parental sleep deprivation. Additional costs that participants noted that were not captured in the survey included insurance premiums or deductibles, the cost of secondary insurances needed, the need for self-employment insurance, moving residences at a financial loss, inability to afford necessary medical equipment (e.g. chair lifts), swimming pool lifts, and nursing costs. Comments varied, with some participants stating financial concerns were not an issue due to adequate insurance, while others reported lifelong struggles with multiple insurance policies along with poor governmental assistance and high deductibles.

#### Responses on HRQoL impact

Of the 12 caregiver participants who provided comments, six (50%) highlighted issues related to HRQoL. Participants noted that the physical impact and intensity of required levels of caregiving was not adequately captured by the survey questions. Participants reported sustaining injuries, an inability to lift their child, and “wear and tear” on their body. This was compounded in participants who were XLMTM carriers or were themselves affected by XLMTM. Participants expressed feelings of helplessness and guilt over the impact of their physical limitations in caring for their children, and fear for their children’s survival.

Participants noted social and societal impacts, such as profound feelings of isolation and loneliness, with the loss of religious communities, friends, and family. One participant noted the impact of not having a co-parent and managing the care responsibilities alone.

All six participants who provided open responses emphasized the emotional impact and stress of their role, describing the impact of living in a “fight-or-flight” state, needing to provide daily life-saving measures, and the constant fear that their child will die.

## Discussion

To our knowledge, this is the first study to describe the financial and health-related impact on caregivers, primarily parents (21 out of 22 participants), who care for individuals with XLMTM. Our results suggest a significant impact on family income in terms of lost employment, missed promotion opportunities, and the need to resign from jobs to be a caregiver and provide the necessary level of care. Parents may have a significant loss of financial income over their lifetime due to extensive years of caregiving, most often unpaid by states or at a national level, and/or a reduction in potential or future income by not being able to pursue higher education and other professional/educational opportunities. Although not evident in our results, the potential higher financial risk for older caregivers compared with their peers from not having had opportunities to pay into employment retirement plans or social security should not be discounted. Although the impact of distractions due to caregiving needs on productivity was evaluated in the survey using absenteeism, the issue of presenteeism was not specifically assessed [20].

Results from this survey also highlight the poor HRQoL of caregivers. The disutility is expressed in relation to the expected average general US population with similar age/sex, and shows a decrease in utility in comparison (i.e. disutility), which is illustrated in Fig. 3. In other words, among the 12 participants who completed the EQ-5D-5L assessment, HRQoL was lower than United States population norms [17] (mean EQ-5D-5L utility index value: 0.656 vs. 0.851; VAS: 71.7 vs. 80.4, respectively, where lower values indicate worse HRQoL). The impact on HRQoL appeared to be related to caregiver age, percentage of unpaid caregiving, caregiving workload (percentage) for each unpaid caregiver, and daily ventilation hours. Older caregivers with a longer duration of caregiving reported poorer HRQoL than younger caregivers with a shorter duration of caregiving. The percentage of unpaid caregiving and the caregiving load per unpaid caregiver also appeared to negatively impact caregiver HRQoL. Individuals with XLMTM are reliant on the use of advanced medical technology or interventions, and as such caregivers may struggle to identify external highly skilled caregivers with adequate skills and experience, especially given the national nurse caregiving shortage [21, 22].

The anxiety experienced by caregivers may be due in part to a constant state of tension resulting from the unpredictable nature of caring for an individual with XLMTM. The impact on anxiety/depression may also be explained by the intensity of caregiving, with little respite, and the lack of support, time, and resources to access activities or social relationships that could help to relieve feelings of social isolation, anxiety, or minimize the impact of depression [23–25]. Additionally, the level of medical complexity, vulnerability, and uncertainty of this rare disease may lead caregivers to have feelings of deep concern, despair or guilt over potential poor outcomes or death, of the individual with XLMTM.

The pain/discomfort reported by caregivers may be attributed to the physical nature of being a caregiver of an individual with a rare disease that has such a profound impact on physical well-being. Often the dependency on caregivers to assist with lifts, transfers, and repositioning may contribute to caregiver injuries or chronic pain [26]. Some repetitive motions or physical tasks may include bed mobility and/or transfers requiring physical heavy lifting particularly for older or bigger individuals with XLMTM. Additional physical strain or difficulties may also be attributed to the caregiver’s experience living with XLMTM themselves if they are a female living with clinical manifestations of XLMTM. Although XLMTM primarily affects males, there is a growing body of research demonstrating that X‑linked diseases can also directly impact females [4]. Previously, females were often only recognized as non-manifesting carriers of X-linked conditions [4].

The physical impact and intensity of the XLMTM caregiver’s experience is in line with perspectives reported by caregivers of patients with other neuromuscular disorders that share a similar symptom profile and prognosis to XLMTM. In a study of families of patients with Duchenne or Becker muscular dystrophies, mothers of patients reported worse HRQoL and emotional distress, including high levels of clinically elevated anxiety symptoms, compared with sex and age group-matched controls [27]. A study by Landfeldt et al. [28] in 2016 reported significant correlations between caregiver anxiety and depression and annual household cost impact, as well as the amount of leisure time used for informal care for individuals with Duchenne muscular dystrophy. Furthermore, spinal muscular atrophy studies conducted in Germany and the Netherlands found a negative correlation between the impact on caregivers and the patients’ motor and functional scores (i.e. lower HRQoL was associated with patients who had less motor abilities) with resultant higher depression and anxiety, fewer leisure activities, and more health impairments (including back, muscle, knee, hip, and digestive pain) of the caregivers [29, 30]. In the current study, the disutility of respondents was particularly marked within the mobility and self-care dimensions: two respondents scored five on mobility and self‑care, indicating they were unable to walk about and unable to wash or dress themselves. Given these low scores, it is possible that participants may have responded to this question with information on the individual with XLMTM, rather than themselves. As such, these 2 values were excluded as outliers. However, the qualitative statements provided by caregivers in the open-ended comments support a level of disutility related to sustaining injuries and chronic stress on the body from caring for individuals with XLMTM, and the possibly impaired physical condition of being affected by clinical manifestations of XLMTM themselves.

There are some limitations to this study. One is that EQ-5D-5L is a general instrument designed to capture HRQoL in any disease area and is not adapted to capture the nuances and depth of the caregiver experience in communities with neuromuscular diseases such as XLMTM. The measures of quality of life used in this assessment may not truly align with each family’s priorities of what is most important to their family and/or child’s lived experience, culture, or values. In addition, the subjective evaluation of quality of life by the caregivers might also not truly reflect the hardship they experience in comparison with the general population due to potential caregiver qualities such as resilience, an optimistic mindset, and dedication to caring for their children with XLMTM [31]. Caregivers may choose to adopt a positive approach as a coping mechanism, and therefore, the full impact of caregiving may not be captured [31]. Grief and loss are difficult to quantify and distinguish from other aspects that may or may not be directly or indirectly related to being a caregiver including family stress, financial stress, health issues, aging, and mental health. Furthermore, a survey study only provides a snapshot in time, and HRQoL is a dynamic process that has many dependent factors over a lifetime.

There is also an element of selection bias in our sample, as parents who choose to be the primary medical caregiver for their children are a select and unique group. We attempted to overcome these limitations by including an open-ended question at the end of the survey so participants could document their specific experiences. While caregivers were overwhelmed by the experience of caregiving, the prospect of it coming to an end due to the death of a child was also terrifying. The complexity of the caregivers’ emotions was difficult to capture in depth: as one participant commented, “it’s important to note that with ALL of this, our son is amazing. He is the light of our lives. We would go broke and live in our van if we needed to, in order to give him his best life.” Taken together, these observations highlight the need for HRQoL instruments that are specifically validated for XLMTM and other neuromuscular diseases.

Other limitations include the small sample size typical of studies of rare diseases and the lower completion rates for the EQ-5D-5L survey (12 families of 22), likely due to the inconvenience of having to leave the main survey site and download a separate document that then needed to be reuploaded. Due to the limited sample size, formal statistical evaluation of several factors was not performed, such as the living status of the individual with XLMTM, the type and the amount of ventilation needed, and net loss of mobility, feeding, and communication skills. Furthermore, the design of the survey, wherein individuals with XLMTM > 25 years of age were categorized in one bracket, prevented full analysis of demographics.

In summary, these real-world data provide a unique opportunity to highlight the significant impact of XLMTM on caregivers. Although a few therapies are in development, there is a clear unmet need for disease modifying treatments. In the absence of such, care is limited to complex supportive medical care. Additionally, the results of this caregiver-reported survey study highlight the need for quality-of-life instruments that are more specifically designed and validated for both XLMTM itself, as well as for caregivers of individuals with complex care needs. Improving comprehensive insurance coverage, increasing supplemental financial assistance, addressing the national caregiving crisis, and advancing national healthcare policies could help to improve the lives of individuals living with XLMTM, their families, and caregivers.

## Supplementary Information

Additional file1

## Data Availability

Details for how researchers may request access to anonymized participant level data, trial level data and protocols from Astellas sponsored clinical trials can be found at https://www.clinicaltrials.astellas.com/transparency/.

## Abbreviations

EQ-5D-5L: EuroQol 5-dimension 5-level
HRQoL: Health-related quality of life
MTM1: Myotubularin 1
VAS: Visual analog scale
XLMTM: X-linked myotubular myopathy
