# Supplementary material for: A real-world analysis of the impact of X-linked myotubular myopathy on caregivers in the United States
Source: Orphanet J Rare Dis. 2025 May 12;20:224. doi: 10.1186/s13023-025-03583-w (PMC12067675; doi:10.1186/s13023-025-03583-w)
Supplement: Supplementary file 1 — Additional file1 [file 13023_2025_3583_MOESM1_ESM.docx]

# Supplementary material

## Supplementary methods

### Cost and health resource survey

The survey consisted of 36 questions formulated by the study group based on advocacy groups and clinician feedback.

#### Care recipient and caregiver characteristics

Care recipient characteristics included age, gender, mobility status (best and current), feeding status (highest and current), speaking/communication status (highest and current), hours and type of ventilation needs, and caregiving needs (paid and unpaid, number of unpaid caregivers). Caregiver characteristics included age, gender, and years of caregiving.

#### Impact on work and caregiver productivity loss

Participants were asked to disclose the impact of their caregiving role on their career. Responses were limited to amount of unpaid leave, the need to resign, reduction in hours, whether a promotion had been refused by the caregiver, and impact on projected career path. Productivity loss for the previous 12 months was estimated using caregiver-reported number of employment hours per week for the entire family. Participant-reported missed working days due to planned or unplanned appointments and any other X-linked myotubular myopathy (XLMTM)-related issues were also taken into account.

#### Out-of-pocket expenses

Out-of-pocket expenses were categorized as either annual or one-time/lifetime costs. Caregivers responded as to whether their related care recipients with XLMTM had that specific cost (e.g., wheelchair, assistive technology/tablets, disposable medical equipment, ventilation, medical procedures, medical therapy, vehicle and/or home modification) and whether it was covered in full, partially, or not at all by insurance. If out-of-pocket costs were incurred, caregivers were asked to estimate the dollar amount in an open-ended response box. The same approach was used for assessing healthcare resource utilization in the past 12 months (not lifetime). Specifically, the number of average monthly visits of a person with XLMTM to healthcare providers or specialists, total hours per week the person with XLMTM traveled to healthcare providers or specialists, and the time the person with XLMTM spent in the hospital during the last 12 months were collected. These expenses included additional vacation expenses, caregiver training, school transportation, and healthcare provider visits that required travel.

#### Financial assistance

Information related to financial assistance was gathered using 2 questions. The first question asked whether they had received assistance through (1) charitable organizations, (2) family and friends, (3) patient advocacy groups, or (4) other sources—with a fill-in option to report the other type of assistance—and if so, how much money in total. The second question asked whether the person with XLMTM received Social Security Disability Income (SSDI) and, if so, the amount received while the caregiver cared for the care recipient with XLMTM.

#### Open-ended qualitative question

At the end of the survey, an open-ended question was included asking caregivers to report any additional information they felt may have been missed by the survey in relation to caregiver HRQoL and additional expenses due to caring for someone with XLMTM. The main themes of these responses were extracted and summarized.

## Supplementary material 1

### Email used to share questionnaire

**Share Your Experience with XLMTM:**

**Family and Caregiver Economic and Quality of Life Impact of XLMTM Study**

Audentes Therapeutics, Inc. recently hired Precision Xtract, an independent consulting company specializing in conducting health outcomes studies, to field a non-interventional (meaning no investigational product is given) study of parents and caregivers of children with XLMTM. This study will collect information about the experiences of XLMTM families and other caregivers in caring for XLMTM patients via an anonymous, internet-based survey. Parents and caregivers of XLMTM patients that are living or that have passed are eligible to participate. The goal of this study is to create a more thorough understanding of the quality of life and economic impact of XLMTM on families and caregivers. Upon completion of the study, the results will be published and shared with the XLMTM community. In addition, summary results could be shared in the future with health policy makers and insurance companies to support patient access to potential treatments.

If you are interested in participating, please follow the link at the bottom of this page. More detailed information about the study, who is eligible to complete the survey and important privacy and consent information are available via the link below.

Your privacy, and that of your child, is of the highest consideration for the conduct of this study and the sharing of its results. At no time will your child’s identity, your identity, or any individual patient or caregiver information be shared publicly, or in any way that can be directly linked to you or your child.

Since you all lead very busy lives, the survey is designed so that it may be started and paused so that it may be completed in multiple sessions on the same laptop/desktop computer, if desired. The entire survey is intended to take no more than one hour in total time to complete. Your support of this study is much appreciated and will provide important insights on the quality of life and economic aspects of XLMTM.

## Supplementary material 2

### Web-Based Survey Tool

*Families and Caregivers Economic and Quality of Life Impact of XLMTM Study*

**Parent/Caregiver Survey Question Outline:**

Introduction:

Thank you for your interest in this study, which will help create a more complete understanding of the impacts of X-linked myotubular myopathy (XLMTM) on the families of children with XLMTM.

The aim of this study is to further characterize the health-related quality of life and economic impact of XLMTM on families and caregivers. Health related quality of life refers to the physical and psychological well-being of parents and caregivers while caring for patients with XLMTM. Economic impact is intended to capture as broadly as possible healthcare costs, family incurred costs, and indirect costs such as caregiver inability to work full time.

This information will be important to support the development of potential new therapies. It will also support analyses and publications that will be requested by insurance companies and other global payers to enable patient access to new therapies.

The survey supporting this study may be completed separately by each parent or caregiver of the person with XLMTM. However, the survey may only be completed once by you for each person with XLMTM that you are caring for.

The survey will take approximately 60 minutes to complete but it **can** be paused and will save so that it can be completed in multiple sittings. Some of the questions will ask about certain quantities and/or costs (e.g. ‘number of times’ or costs), but please do not worry if you cannot remember these figures exactly; just provide your best estimate.

You are eligible to complete this survey if you are 18 years of age or older and you are a parent or caregiver of a person with XLMTM, either currently living or that has passed away.

If you are a person with XLMTM over the age of 18, you are not eligible to complete this current survey. However, you may be eligible for future studies on this topic. If you would like to be contacted about future surveys, please provide your e-mail address here: (program e-mail capture).

The survey results will be analyzed by an independent consultant (Precision Xtract) who has signed a binding confidentiality agreement. Only Precision Xtract will have access to information that could potentially identify individual patients and will have access only for the purposes of collecting and aggregating data for this specific survey. Aggregated responses will be consolidated and shared in a way that preserves patient confidentiality and only summaries of the responses will be shared with the study sponsor, Audentes Therapeutics, Inc., and included in confidential regulatory submissions. Final aggregated results are also planned to be submitted for publication in a peer-reviewed scientific journal to broaden publicly available knowledge about XLMTM. You will be asked at the beginning of the survey whether you consent to being part of this study.

**The survey is sponsored by Audentes and approved by the Advarra Institutional Review Board to be used for research purposes.**

If you have any difficulties accessing this survey, please contact ivar.jensen@precisionxtract.com.

We are very grateful for your time and insights.

The closing date for this survey is 4 months post fielding.

Thank You.

Consent:

Aggregated responses may be shared with insurance companies, health technology assessors representing payers, Audentes Therapeutics, Inc. (and its agents and representatives), and/or included in confidential regulatory submissions for the evaluation of potential new XLMTM therapies. Final aggregated results are also planned to be submitted for publication in a peer-reviewed scientific journal to broaden publicly available knowledge about XLMTM. In no instances will these aggregated responses contain any information that will identify individual patients.

No information that could lead to identification of individuals will be provided to any of the permitted recipients described above. At no point will Audentes (or its agents or representatives) or Precision Xtract purchase, use or sell any identifying personal information provided in the survey. Electronically stored identifying information will not be shared publicly but will only be captured and stored by Precision Xtract to allow respondents to pause, save, and/or retake the survey. Upon completion of the analysis of survey results, any potential identifying electronic information, such as geolocation or IP addresses, will be deleted.

Do you consent to our use of the information you provide in this survey?

- **YES**
- **NO [Programming note: Exclude from study if selected]**

Eligibility:

1. In which country do you currently live?

- United States
- Other (please specify):
  [TEXT FIELD]

1. XLMTM predominantly affects boys, however, we acknowledge some girls and women are also affected by XLMTM. Please tell us the gender at birth of the person with XLMTM that you will be answering questions about.

- Male
- Female
- Prefer not to answer

1. Is the person with XLMTM currently enrolled in the investigational clinical trial ASPIRO?

- YES
- NO

1. Do you know if there are other parents or caregivers filling out a survey for this child?
   - YES
   - NO
2. How old is the person with XLMTM that you will be answering questions about?

- <1 year old
- [ NUMBER FIELD] years old (Whole number ages)
- Deceased
- Prefer not to answer

1. How many years ago did the person with XLMTM pass away? *[Programming note: If “Deceased” selected for Q4]*

- Less than or equal to 2 years ago
- More than 2 years ago
- Prefer not to answer

1. How old were they when they passed away? *[Programming note: If “Deceased” selected for Q4]*

- [ AGE DROPDOWN] {Programming note: Whole number ages will be presented starting from “<1 year old”]
- Prefer not to answer

***Text in purple signifies text to be utilized if parents/caregivers select “Deceased” for Question 4. If the child is living, then the purple text will be omitted from all questions. ***

1. Please tell us who is completing this survey:

- I am a parent (age 18 years or older) of a child with XLMTM
- I am an unpaid caregiver (e.g., sibling, grandparent, etc.) (age 18 years or older) of a child with XLMTM
- I am a paid caregiver (i.e., a person who is hired to care for a child with XLMTM, such as a nurse or another individual paid for by a family, insurer, government program, or other organization) (age 18 years or older) of a child with XLMTM
- I am a patient with XLMTM (age 18 years or older) [Because you are an XLMTM patient greater than the age of 18, you may be eligible for future studies on this topic. If you would like to be contacted about such a survey in the future, please provide your e-mail address here]
- Other (please specify): [TEXT FIELD]

1. For how many years have you cared for the person with XLMTM?

Please specify: [ NUMBER FIELD] Years

Demographics:

1. What is your age?

[ AGE DROPDOWN] (Whole number ages will be presented starting from “<18 years old”)

[participants <18 years old will be excluded from the study]

1. What is your gender?

- Male
- Female
- Other
- Prefer not to answer

1. Please select the highest level of education of the primary household earner?

- No High School
- Some High School
- Completed High School/GED or High School equivalent
- Bachelor’s Degree
- Master’s Degree
- Doctorate
- Other (please specify):
  [ TEXT FIELD]

Health-Related Quality of Life:

To better understand how XLMTM impacts not only those with the condition, but also parents and caregivers, please answer the following questions about **your** mental and physical health.

Under each heading, please tick the ONE box that best describes your health TODAY.

**MOBILITY**

- I have no problems in walking about
- I have slight problems in walking about
- I have moderate problems in walking about
- I have severe problems in walking about
- I am unable to walk about

**SELF-CARE**

- I have no problems washing or dressing myself
- I have slight problems washing or dressing myself
- I have moderate problems washing or dressing myself
- I have severe problems washing or dressing myself
- I am unable to wash or dress myself

**USUAL ACTIVITIES** (e.g. work, study, housework, family or leisure activities)

- I have no problems doing my usual activities
- I have slight problems doing my usual activities
- I have moderate problems doing my usual activities
- I have severe problems doing my usual activities
- I am unable to do my usual activities

**PAIN/DISCOMFORT**

- I have no pain or discomfort
- I have slight pain or discomfort
- I have moderate pain or discomfort
- I have severe pain or discomfort
- I have extreme pain or discomfort

**ANXIETY/DEPRESSION**

- I am not anxious or depressed
- I am slightly anxious or depressed
- I am moderately anxious or depressed
- I am severely anxious or depressed
- I am extremely anxious or depressed

**
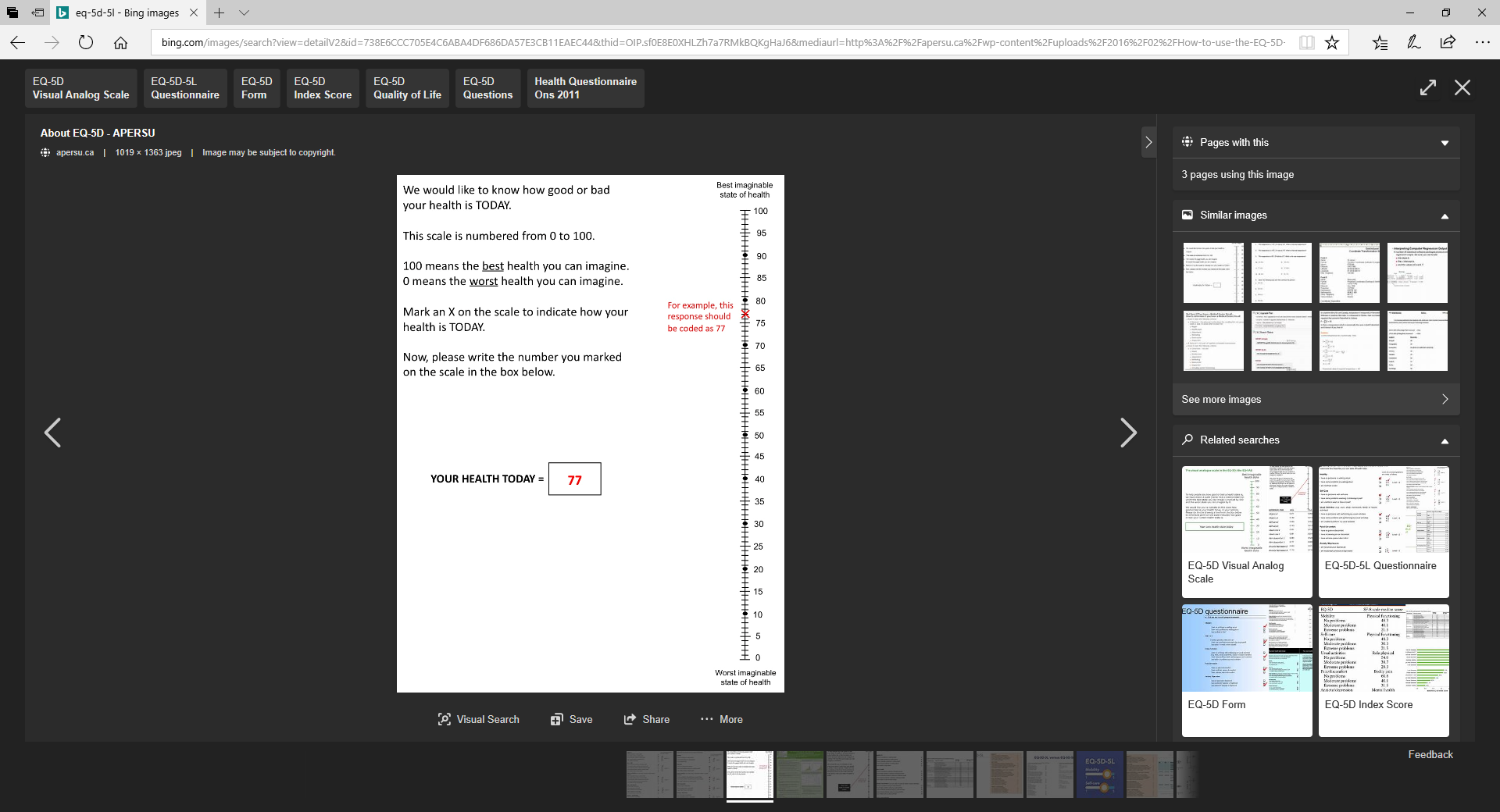
**

**We would like to know how good or bad your health is TODAY.**

**This scale is numbered from 0 to 100.**

**100 means best heath you can imagine.**

**0 means the worst health you can imagine.**

**Please click on the scale to indicate how your health is today.**

**Your Health TODAY = 77**

Patient Clinical Information:

To better understand how XLMTM impacts the financial cost to families caring for children with XLMTM, please answer the following question about the person with XLMTM.

1. Does the person with XLMTM currently/In the last 12 months before the person with XLMTM passed away, did the person with XLMTM require any mechanical ventilator support?

- YES
- NO

*[Programming note: If YES on Question 13 show Question 14]*

1. Does the person with XLMTM currently/In the last 12 months before the person with XLMTM passed away, did the person with XLMTM require any of the following types of ventilator support (Please select all that apply)? If so, how many hours per day, on average, does/did the person with XLMTM require this ventilator support?

- BIPAP (Bilevel Positive Airway Pressure)
  - [ NUMBER FIELD] Hours per day
- CPAP (Continuous Positive Airway Pressure)
  - [ NUMBER FIELD] Hours per day
- IPPV (Intermittent Positive-Pressure Ventilation)
  - [ NUMBER FIELD] Hours per day
- Pressure Support
  - [ NUMBER FIELD] Hours per day
- SIMV (Synchronized Intermittent Mechanical Ventilation)
  - [ NUMBER FIELD] Hours per day
- Supplemental Oxygen
  - [ NUMBER FIELD] Hours per day
- Invasive Ventilation (e.g. ventilation via tracheostomy)
  - [ NUMBER FIELD] Hours per day
- Other (please specify):
  - [ NUMBER FIELD] Hours per day

1. Considering the person with XLMTM, which of the following motor function abilities most closely describes their best achieved state of mobility over their lifetime?

- Unable to sit
- Sitting without support but cannot roll
- Sitting with support
- Sitting and rolling independently
- Sitting and crawling/bottom shuffling with hands and knees
- Standing and walking with assistance
- Standing unaided
- Walking unaided

1. Considering the person with XLMTM, which of the following motor function abilities most closely describes their current state of mobility/state of mobility in the last 12 months before the person with XLMTM died?

- Unable to sit
- Sitting without support but cannot roll
- Sitting with support
- Sitting and rolling independently
- Sitting and crawling/bottom shuffling with hands and knees
- Standing and walking with assistance
- Standing unaided
- Walking unaided

1. Considering the person with XLMTM, which of the following motor function abilities most closely describes their best achieved state of ability when eating over their lifetime?

- Feeding self with no difficulties
- Feeding self with parental/caregiver support or feeding assistive devices
- Requiring a nasogastric or gastric tube (i.e., NG-Tube or G-Tube and/or suction machine)

1. Considering the person with XLMTM, which option below would most closely describe their current abilities when eating/abilities when eating in the last 12 months before the person with XLMTM passed away?

- Able to feed self with no difficulties /Fed self with no difficulties
- Able to feed self with parental/caregiver support or feeding assistive devices/Fed self with parental/caregiver support or feeding assistive devices
- Requires/Required a nasogastric or gastric tube (i.e., NG-Tube or G-Tube and/or suction machine)

1. Considering the person with XLMTM, which of the following motor function abilities most closely describes their best achieved state of ability when speaking/communicating vocally?

- Speaking (i.e., clearly vocalize words) with no difficulties
- Speaking (i.e., clearly vocalize words) with some difficulties
- Communicating with sign language
- Communicating with assistive communication devices
- Communicating using a speaking valve with a tracheostomy
- Unable to speak or communicate
- Other (please specify):
  [TEXT FIELD]

1. Considering the person with XLMTM, which option below would most closely describe their current abilities when speaking/communicating vocally/abilities when speaking/communicating vocally in the last 12 months before the person with XLMTM passed away?

- Ability to speak (i.e., clearly vocalize words) with no difficulties /Spoke with no difficulties
- Ability to speak (i.e., clearly vocalize words) with some difficulties /Spoke with some difficulties
- Able/Was able to communicate with sign language
- Able/Was able to communicate with assistive communication devices
- Able/Was able to communicate using a speaking valve with a tracheostomy
- Unable/Was unable to speak or communicate
- Other (please specify):
  [TEXT FIELD]

Unpaid Caregivers:

1. Each week, on average, how many people (e.g., partner, sibling, grandparent, etc.) are/were involved in unpaid care of the person with XLMTM (in addition to yourself)?

[NUMBER FIELD DROPDOWN] (if 0 is selected skip to Q25)

1. Each week, on average, how many total hours of unpaid caregiver (e.g., partner, sibling, grandparent, etc.) time is/was provided to caring for the person with XLMTM (include any travel time, medical visits and nighttime care, if applicable)?

Caregivers: [NUMBER FIELD] Hours

1. Has/Had any unpaid caregiver of the person with XLMTM needed to reduce hours worked in paid employment (including yourself)?

- Yes
- No

1. Please fill out the following questions below if any unpaid caregiver(s) of the person with XLMTM (including yourself) have needed to reduce their hours worked in paid employment, give up paid employment entirely or refuse a promotion/change career goals. Please assume 40 hours per week is full time.

|  | *How many total hours per week have unpaid caregivers of the person with XLMTM had to reduce their other working hours by?* | *Please check the box if an unpaid caregiver of the person with XLMTM needed to give up paid employment completely?* | *Please check the box if an unpaid caregiver of the person with XLMTM needed to refuse promotion or change career goals?* |
| --- | --- | --- | --- |
| Caregivers: | [ NUMBER FIELD] Hours | [ CHECK BOX] | [ CHECK BOX] |

1. Over the last 12 months/In a typical year, how often have/had you missed or called out of work for:

Planned appointments? [NUMBER FIELD] Full Days; [NUMBER FIELD] Half Days

Unplanned appointments? [NUMBER FIELD] Full Days; [NUMBER FIELD] Half Days

Other XLMTM-related issues (e.g., sudden ill-health, sick leave due to psychic stress, equipment breakdown, etc.)?
 [NUMBER FIELD] Full Days; [NUMBER FIELD] Half Days

Paid Caregivers:

1. Each week, on average, how many total hours of paid caregiver time is/was provided to caring for the person with XLMTM at home or at school? (Include any night time care, if applicable)

Caregivers: [ NUMBER FIELD ] Hours

Medical Resource Use:

1. Does the person with XLMTM currently or ever/Did the person with XLMTM, in the 12 months before passing away or ever, have this equipment/intervention, and did the family pay the full cost or part of the cost (i.e. did insurance, patient assistance groups, government payers, etc. pay part of the cost)?

| *Equipment/Intervention:* | *Has/had the person with XLMTM ever have this equipment/ intervention over their lifetime?* | *Does/Did the person with XLMTM currently/, in the 12 months before passing away, have this equipment/intervention?* | *How was the equipment paid for?* | *(If Family paid Full or Part:)*  *What would you estimate the family’s total out of pocket expenses to be for the time the family cared for the child with XLMTM?* |
| --- | --- | --- | --- | --- |
| Wheelchairs (e.g., Powered wheelchair, manual wheelchair, specialist buggy, Wizzybug, etc.) | [Yes/No/Not sure dropdown] | [Yes/No/Not sure dropdown] | [Family paid full (All costs)/Part (Insurance paid some and family paid some)/Family paid none/Not sure dropdown] | [$] |
| Assistive Technology (e.g., Standing/walking frame, specialist seating, orthotics, mobile arm supports, lycra suit, spinal brace/jacket, iPad, assisted communication devices, etc.) | [Yes/No/Not sure dropdown] | [Yes/No/Not sure dropdown] | [Family paid full (All costs)/Part (Insurance paid some and family paid some)/ Family paid none/Not sure dropdown] | [$] |
| Disposable Medical Equipment (e.g., tubing, connectors, diapers, eye drops, gels, etc.) | [Yes/No/Not sure dropdown] | [Yes/No/Not sure dropdown] | [Family paid full (All costs)/Part (Insurance paid some and family paid some)/ Family paid none/Not sure dropdown] | [$] |
| Ventilation (e.g., cough assist machine, nebulizer, day/night time non-invasive ventilation, mechanical ventilation, tracheostomy, etc.) | [Yes/No/Not sure dropdown] | [Yes/No/Not sure dropdown] | [Family paid full (All costs)/Part (Insurance paid some and family paid some)/ Family paid none/Not sure dropdown] | [$] |
| Medical Procedure (e.g., spinal rods/spinal fusion, postural draining, gastrostomy, etc.) | [Yes/No/Not sure dropdown] | [Yes/No/Not sure dropdown] | [Family paid full (All costs)/Part (Insurance paid some and family paid some)/ Family paid none/Not sure dropdown] | [$] |
| Medical Therapy (e.g., chest physiotherapy, hydrotherapy, etc.) | [Yes/No/Not sure dropdown] | [Yes/No/Not sure dropdown] | [Family paid full (All costs)/Part (Insurance paid some and family paid some)/ Family paid none/Not sure dropdown] | [$] |
| Home Modification (e.g., specialist bed/mattress/sleep system, adaptations to bathroom/kitchen, ramp installations, etc.) | [Yes/No/Not sure dropdown] | [Yes/No/Not sure dropdown] | [Family paid full (All costs)/Part (Insurance paid some and family paid some)/ Family paid none/Not sure dropdown] | [$] |
| Vehicle Modification (e.g., specialist car seat, wheelchair accessible vehicle, etc.) | [Yes/No/Not sure dropdown] | [Yes/No/Not sure dropdown] | [Family paid full (All costs)/Part (Insurance paid some and family paid some)/ Family paid none/Not sure dropdown] | [$] |
| Other (please specify) |  |  |  |  |

Healthcare Provider Visits:

1. On average, how many times per month do/did the person with XLMTM visit their healthcare provider(s) and/or specialist(s)?

[NUMBER FIELD] visits per month

1. In total, how many hours per week does/did the person with XLMTM have to travel to see their healthcare provider(s) and/or specialist(s) in terms of travel time?

- Less than 1 hour per week
- Less than 2 hours per week
- Less than 3 hours per week
- Less than 4 hours per week
- Less than 5 hours per week
- Less than 6 hours per week
- More than 6 hours per week: [NUMBER FIELD] hours of travel time

1. In the last 12 months/In the 12 months before passing away, how much time has/had the person with XLMTM spent in the hospital?

[ DROPDOWN] (Counts weeks up to 4 weeks starting with “less than 1 week per year” and then counts months up to “more than 11 months per year”)

1. In the last 12 months/In the 12 months before passing away, how much money ($) have/had all caregivers (paid and unpaid caregivers) and the person with XLMTM spent on healthcare provider, specialist and hospital visits related to travel, parking and overnight stays (if appropriate)?

Please specify (whole number):

Total ($) [ NUMBER FIELD] per year

1. In the last 12 months/In the 12 months before passing away, how much money ($) have/had you or the person with XLMTM spent on:

- A specialist school for the person with XLMTM (if applicable):
  Total ($) [ NUMBER FIELD] per year
- Transportation of the person with XLMTM to and from school:

Total ($) [ NUMBER FIELD] per year

- XLMTM caregiver training sessions for parents and/or caregivers:

Total ($) [ NUMBER FIELD] per year

- Additional vacation/holiday expenses (i.e., additional expenses required to travel with and accommodate caregivers):

Total ($) [ NUMBER FIELD] per year

1. Have you or the person with XLMTM received financial assistance from any of the following? (Please select all that apply)

- Charitable organizations
- Family and friends
- Patient advocacy groups
- Other (Please Specify)
  1. [If 1-4 are checked] What would you estimate the family’s total financial assistance to be for the time the family cared for the child with XLMTM?

1. Is/Was the person with XLMTM receiving Social Security Disability Income?

- Yes
- No
- Do not know
  1. [If yes] What would you estimate the family’s total Social Security Disability Income to be for the time the family cared for the child with XLMTM?

1. Is there anything else that you would like to share about your experiences with XLMTM with respect to caring for an XLMTM patient, family impacts, quality of life or economics that were not covered by the survey? (Allow for open-ended response below)
2. Do you agree to being contacted confidentially in the future by a third-party company representing Audentes if any additional non-interventional research study opportunities arise?

- YES
- NO

**Thank you for taking the time to take part in this survey.**

**If you have any questions, please contact: ivar.jensen@precisionxtract.com**

**If you would like us to send you the results, please enter your email address below:**

Supplementary Table S1 Characteristics of the caregiver participants and individual with XLMTM and impact of caregiving on caregiving participants

| **Participant number** | 1 | 2 | 3 | 4 | 5 | 6 | 7 | 8 | 9 | 10 | 11 | 12 | 13 | 14 | 15 | 16 | 17 | 18 | 19 | 20 | 21 | 22 |
| --- | --- | --- | --- | --- | --- | --- | --- | --- | --- | --- | --- | --- | --- | --- | --- | --- | --- | --- | --- | --- | --- | --- |
| **Caregiver** | | | | | | | | | | | | | | | | | | | | | | |
| Caregiver age | 28 | 24 | 38 | 44 | 34 | 43 | 39 | 42 | 65 | 45 | 35 | 39 | 45 | 50 | 47 | 49 | 39 | 52 | 55 | 58 | 50 | 62 |
| Sex | F | F | F | M | F | F | M | F | F | F | F | F | M | F | F | M | F | F | F | F | F | M |
| Caregiving years | <1 | 1 | 18**^#^ | 3* | 4 | 6 | 6 | 7 | 6 | 11 | 11 | 16 | 18 | 18 | 18 | 22 | 23 | 24** | 24 | >25 | >25 | >25 |
| Highest level of education | Other | Other | High school  /GED | Bachelor  degree | Bachelor  degree | Master’s  Degree | Bachelor  degree | Bachelor  degree | Other | Bachelor  degree | Other | Master’s  Degree | Bachelor  degree | Master’s  Degree | High school  /GED | Master’s  Degree | Bachelor  degree | Bachelor  degree | Bachelor  degree | Bachelor  degree | Master’s  Degree | Master’s  Degree |
| **Productivity** | | | | | | | | | | | | | | | | | | | | | | |
| Additional unpaid caregiver number | 5 | 1 | 2 | 3 | 4 | 0 | 5 | 1 | 2 | 2 | 3 | 2 | 1 | 2 | 1 | 1 | 5 | 2 | 0 | 2 | 0 | 2 |
| % unpaid caregiving | 100% | 46% | 69% | 45% | 48% | 0 | 74% | 44% | 16% | 11% | 63% | 68% | 50% | 27% | 60% | 46% | 33% | 48% | 0 | 50% | 0 | 33% |
| Family lost 1 full income – refuse promotion or change career | Y/Y | Y/– | –/Y | Y/– | –/– | –/– | Y/– | Y/Y | –/– | –/Y | –/Y | –/– | –/Y | –/Y | –/– | –/– | –/– | Y/Y | –/– | Y/Y | –/– | Y/– |
| Total caregiver employment reduction/ week/family | 40 h | 40 h | 20 h | 50 h | – | – | 10 h | 10 h | 10 h | 24 h | 20 h | 5 h | 40 h | – | 30 h | – | – | 20 h | – | 30 h | – | 40 h |
| Caregiver missed workdays  planned appt (past 12 mo) | – | 2 | 3 | 30 | 30 | – | 51.5 | 15 | 2 | 16 | 4 | 16.5 | 7.5 | 1 | 10 | 2 | 2 | 45 | – | – | – | 5 |
| Caregiver missed workdays unplanned appointment (past 12 mo) | – | 4 | 2.5 | 15 | 4.5 | – | 8 | 10 | 0 | 6 | 1 | 7.5 | 0.5 | 0 | 0 | 2 | 0 | 35 | – | – | – | 1.5 |
| Caregiver missed workdays XLMTM related issue (past 12 mo) | – | 4 | 4 | 3.5 | 0 | – | 2 | 15 | 0 | 70 | 4 | 10 | 7 | 0 | 5 | 0 | 6 | 52.5 | – | – | – | 4 |
| **Estimated productivity loss/y ($1000)** | **62.1** | **64.5** | **33.3** | **89.2** | **8.2** | **0** | **30.2** | **25.1** | **16.0** | **59.2** | **33.2** | **15.9** | **65.7** | **0.2** | **50.2** | **1.0** | **2.6** | **62.7** | **0** | **46.6** | **0** | **64.6** |
| **Individual with XLMTM** | | | | | | | | | | | | | | | | | | | | | | |
| Age | <1 | 1 | 2** | 3* | 4 | 6 | 6 | 7 | 10 | 11 | 11 | 16 | 18 | 18 | 18 | 22 | 23 | 24** | 24 | >25 | >25 | >25 |
| Invasive ventilation (h/day) | 24 | 24 | 24 | – | – | 24 | 24 | 24 | 24 | 24 | 24 | – | 24 | 24 | – | – | – | 24 | 24 | – | – | 24 |
| BIPAP (h/day) | – | – | – | 12 | – | 8 | – | – | – | – | – | – | – | - | 24 | 10 | – | – | – | 10 | 24 | – |
| Supportive O_2_ (h/day) | – | – | 18 | 2 | – | 8 | 1 | – | – | – | – | – | 6 | – | – | – | – | 24 | – | – | – | – |
| SIMV (h/day) | – | 24 | – | – | 18 | 16 | – | – | – | – | – | 24 | – | 24 | – | – | – | – | 24 | – | – | – |
| Pressure support (h/day) | 24 | – | 24 | – | 6 | – | 24 | – | 24 | – | 24 | – | – | – | – | – | 24 | – | – | – | – | – |
| IPPV (h/day) | – | – | – | – | – | – | – | – | 24 | – | – | – | – | - | – | – | – | – | – | – | – | – |

Estimated productivity loss/year = (median hourly wage $29.86 in 2023) x (total employment hours reduction/week/family) x 52 + (median hourly pay rate $29.86 in 2023) x (days missed related to XLMTM) x 8

*Died <2 years ago. **Died >2 years ago. #This result is an outlier and unable to be confirmed. The use of a dash indicates that no response was provided.

Abbreviations: BIPAP, bilevel positive airway pressure; F, female; h, hour; IPPV, intermittent positive-pressure ventilation; M, male; mo, month; SIMV, synchronized intermittent mandatory ventilation; XLMTM, X-linked myotubular myopathy; y, year.

Supplementary Table 2 Participant-level caregiver-reported characterization of (A) motor, (B) feeding, and (C) speaking milestones

1. Motor milestones

|  | Participant number | 1 | 2 | 3 | 4 | 5 | 6 | 7 | 8 | 9 | 10 | 11 | 12 | 13 | 14 | 15 | 16 | 17 | 18 | 19 | 20 | 21 | 22 |
| --- | --- | --- | --- | --- | --- | --- | --- | --- | --- | --- | --- | --- | --- | --- | --- | --- | --- | --- | --- | --- | --- | --- | --- |
| Highest Achieved Milestone | Unable to sit |  | x |  |  |  |  | x |  |  | x |  | x |  |  |  |  |  |  |  | x |  |  |
|  | Sitting without support but cannot roll |  |  |  |  | x | x |  |  | x |  | x |  |  |  |  |  |  |  |  |  |  |  |
|  | Sitting with support |  |  | x | x |  |  |  | x |  |  |  |  |  | x | x | x | x |  | x |  | x | x |
|  | Sitting and rolling independently |  |  |  |  |  |  |  |  |  |  |  |  | x |  |  |  |  |  |  |  |  |  |
|  | Walking unaided | x |  |  |  |  |  |  |  |  |  |  |  |  |  |  |  |  | x |  |  |  |  |
| Current Milestone | Unable to sit |  | x |  |  |  |  | x |  | x | x |  | x |  |  |  |  | x |  |  | x |  | x |
|  | Sitting without support but cannot roll | x |  |  |  |  |  |  |  |  |  |  |  |  |  | x |  |  |  |  |  |  |  |
|  | Sitting with support |  |  | x | x | x | x |  | x |  |  | x |  |  | x |  | x |  |  | x |  | x |  |
|  | Sitting and rolling independently |  |  |  |  |  |  |  |  |  |  |  |  | x |  |  |  |  |  |  |  |  |  |
|  | Walking unaided |  |  |  |  |  |  |  |  |  |  |  |  |  |  |  |  |  | x |  |  |  |  |

Some respondents (marked in red) reported better motor milestones currently than their best achieved milestone.

1. Feeding milestones

|  | Participant number | 1 | 2 | 3 | 4 | 5 | 6 | 7 | 8 | 9 | 10 | 11 | 12 | 13 | 14 | 15 | 16 | 17 | 18 | 19 | 20 | 21 | 22 |
| --- | --- | --- | --- | --- | --- | --- | --- | --- | --- | --- | --- | --- | --- | --- | --- | --- | --- | --- | --- | --- | --- | --- | --- |
| Highest Achieved Milestone | Feeding self with no difficulties | x |  |  |  |  |  |  |  |  |  |  |  |  |  |  |  |  |  |  |  |  |  |
|  | Feeding self with parental/caregiver support or feeding assistive devices |  |  |  |  |  |  |  |  |  |  |  |  |  |  |  |  |  |  | x |  |  |  |
|  | Requiring a nasogastric or gastric tube |  | x | x | x | x | x | x | x | x | x | x | x | x | x | x | x | x | x |  | x | x | x |
| Current Milestone | Feeding self with no difficulties | x |  |  |  |  |  |  |  |  |  |  |  |  |  |  |  |  |  |  |  |  |  |
|  | Feeding self with parental/caregiver support or feeding assistive devices |  |  |  |  |  |  |  |  |  |  |  |  |  |  |  |  |  | x |  |  |  |  |
|  | Requiring a nasogastric or gastric tube |  | x | x | x | x | x | x | x | x | x | x | x | x | x | x | x | x |  | x | x | x | x |

Some respondents (marked in red) reported better motor milestones currently than their best achieved milestone.

1. Speaking milestones

|  | Participant number | 1 | 2 | 3 | 4 | 5 | 6 | 7 | 8 | 9 | 10 | 11 | 12 | 13 | 14 | 15 | 16 | 17 | 18 | 19 | 20 | 21 | 22 |
| --- | --- | --- | --- | --- | --- | --- | --- | --- | --- | --- | --- | --- | --- | --- | --- | --- | --- | --- | --- | --- | --- | --- | --- |
| Highest Achieved Milestone | Speaking (i.e., clearly vocalize words) with no difficulties |  |  |  |  |  |  |  |  | x |  |  |  |  |  |  |  |  |  |  |  |  |  |
|  | Speaking (i.e., clearly vocalize words) with some difficulties | x |  | x |  | x | x |  |  |  |  |  | x |  |  | x | x | x | x |  |  |  | x |
|  | Communicating with sign language |  |  |  |  |  |  |  |  |  |  | x |  | x |  |  |  |  |  |  | x |  |  |
|  | Communicating with assistive communication devices |  |  |  | x |  |  | x |  |  |  |  |  |  |  |  |  |  |  |  |  | x |  |
|  | Communicating using a speaking valve with a tracheostomy |  |  |  |  |  |  |  |  |  |  |  |  |  |  |  |  |  |  |  |  |  |  |
|  | Unable to speak or communicate |  |  |  |  |  |  |  |  |  | x |  |  |  | x |  |  |  |  |  |  |  |  |
|  | Other |  | x |  |  |  |  |  | x |  |  |  |  |  |  |  |  |  |  | x |  |  |  |
| Current Milestone | Speaking (i.e., clearly vocalize words) with no difficulties |  |  |  |  |  |  |  |  |  |  |  |  |  |  |  |  |  |  |  |  |  |  |
|  | Speaking (i.e., clearly vocalize words) with some difficulties |  |  |  |  | x | x |  |  |  |  |  | x |  |  | x | x | x | x |  |  |  | x |
|  | Communicating with sign language |  |  |  |  |  |  |  |  |  |  | x |  | x |  |  |  |  |  |  |  |  |  |
|  | Communicating with assistive communication devices |  |  |  | x |  |  | x |  |  |  |  |  |  |  |  |  |  |  |  |  | x |  |
|  | Communicating using a speaking valve with a tracheostomy | x |  |  |  |  |  |  |  | x |  |  |  |  |  |  |  |  |  |  |  |  |  |
|  | Unable to speak or communicate |  |  |  |  |  |  |  |  |  | x |  |  |  | x |  |  |  |  |  | x |  |  |
|  | Other |  | x | x |  |  |  |  | x |  |  |  |  |  |  |  |  |  |  | x |  |  |  |

Supplementary Table 3 Out-of-pocket costs incurred by caregivers of individuals with XLMTM, including one-time expenses, and expenses within the past 12 months

| **Participant number** | 1 | 2 | 3 | 4 | 5 | 6 | 7 | 8 | 9 | 10 | 11 | 12 | 13 | 14 | 15 | 16 | 17 | 18 | 19 | 20 | 21 | 22 |
| --- | --- | --- | --- | --- | --- | --- | --- | --- | --- | --- | --- | --- | --- | --- | --- | --- | --- | --- | --- | --- | --- | --- |
| **Age of son with XLMTM (years)** | <1 | 1 | 2* | 3 * | 4 | 6 | 6 | 7 | 10 | 11 | 11 | 16 | 18 | 18 | 18 | 22 | 23 | 24* | 24 | >25 | >25 | >25 |
| **Direct medical out-of-pocket costs** | | | | | | | | | | | | | | | | | | | | | | |
| Wheelchair ($1000) | – | 1 | + | + | - | + | 4 | 15 | + | + | + | + | + | + | + | + | + | 30 | 5 | + | 2.5 | 5 |
| Assistive Technology ($1000) | 0.1 | 1 | + | + | 3 | + | 1 | 18 | + | 4.5 | .35 | 1 | 2.5 | + | + | + | + | 5 | 25 | 10 | 1.5 | 20 |
| Disposable medical equipment ($1000) | 2 | 0.5 | + | 4 | 2 | 1 | 15 | 5 | + | 30 | 0.1 | 1 | 3 | + | 5 | + | 55 | + | 100 | + | 10 | 300 |
| Ventilation ($1000) | + | + | + | + | 0.3 | 1 | + | + | + | + | + | + | + | + | + | + | + | + | 10 | 20 | + | 5 |
| Medical  Procedures ($1000) | + | + | + | + | + | + | 4 | + | + | + | + | + | + | + | 5 | + | + | 20 | + | 5 | + | 5 |
| Medical therapy ($1000) | 0.1 | + | + | + | 0.5 | + | 20 | + | + | 2.5 | + | + | + | 10 | + | + | + | + | 50 | 5 | + | 1 |
| **Total direct medical ($1000)** | **2.2** | **2.5** | **0** | **4** | **5.8** | **2** | **44** | **38** | **0** | **37** | **0.45** | **2** | **5.5** | **10** | **10** | **0** | **55** | **55** | **190** | **40** | **14** | **336** |
| **Annual direct medical ($1000)** | **0** | **5.4** | **0.6** | **7.3** | **13.4** | **2.4** | **0.6** | **0.3** | **7.9** | **2.5** | **0.6** | **0** | **1.5** | **2.2** | **1.3** | **0** | **2.3** | **1.6** | **3.4** | **0** | **0.3** | **0.1** |
| **Indirect medical out-of-pocket costs** | | | | | | | | | | | | | | | | | | | | | | |
| Home modifications ($1000) | 2 | – | 0.6 | – | 5 | + | 50 | 30 | 5 | 50 | 3 | 20 | 10 | 9 | + | 1 | 10 | 5 | + | 50 | 150 | 25 |
| Vehicle modifications ($1000) | 2 | – | 0.2 | – | 60 | 40 | 180 | 70 | 40 | 25 | 7 | 50 | 130 | 120 | + | 43 | – | 30 | 50 | 60 | 80 | 70 |
| **Total indirect medical ($1000)** | **4** | **0** | **0.8** | **0** | **65** | **40** | **230** | **100** | **45** | **75** | **10** | **70** | **140** | **129** | **0** | **44** | **10** | **35** | **50** | **110** | **230** | **95** |
| **Annual indirect medical ($1000)** | **2.0** | **14.3** | **7.2** | **38.3** | **3.3** | **0.4** | **0** | **6.7** | **2.5** | **0** | **9.2** | **7.5** | **16.3** | **4.0** | **0** | **0.9** | **1.5** | **4.8** | **6.8** | **0.0** | **7.8** | **4.4** |
| **Total costs** | | | | | | | | | | | | | | | | | | | | | | |
| **Total costs ($1000)** | **6.2** | **2.5** | **0.8** | **4** | **70.8** | **42** | **274** | **138** | **45** | **112** | **10.45** | **72** | **145.5** | **139** | **10** | **44** | **65** | **90** | **240** | **150** | **244** | **431** |
| **Total annual costs ($1000)** | **12.4** | **2.5** | **0.4** | **1.3** | **17.7** | **7** | **45.7** | **19.7** | **4.5** | **10.2** | **1.0** | **4.5** | **8.1** | **7.7** | **0.6** | **2.0** | **2.8** | **3.8** | **10** | **5.8** | **9.4** | **14.4** |
| **1-year expenses** | | | | | | | | | | | | | | | | | | | | | | |
| Additional vacation expenses ($1000) | – | 0.3 | 0.4 | 0.3 | – | – | 2 | – | – | – | 0.5 | – | 1.5 | – | 2 | – | 2 | – | – | – | 1 | 1 |
| Caregiver training ($1000) | – | – | – | – | – | – | 0.1 | – | – | – | – | – | - | – | - | – | – | – | – | – | – | 1.2 |
| School transportation ($1000) | – | – | – | – | – | – | 0.5 | – | – | – | – | – | 0.25 | – | - | – | 1.5 | - | – | – | – | – |
| HCP visits with travel ($1000) | 1.5 | 0.6 | 1.1 | 0.5 | 0.75 | 0.5 | 5 | 0.4 | 5 | 0.35 | 0.3 | – | 0.75 | – | .1 | – | 0.2 | 2.5 | 0.5 | 1 | 1 | – |
| **Total ($1000)** | **1.5** | **0.9** | **1.5** | **0.8** | **0.75** | **0.5** | **7.6** | **0.4** | **5** | **0.35** | **0.8** | **0** | **2.5** | **0** | **2.1** | **0** | **3.7** | **2.5** | **0.5** | **1** | **2** | **2.2** |

*Individual with XLMTM was deceased. + indicates all costs were paid by insurance. - indicates not applicable/no cost occurred. Bolded indicates all expenses were paid by the family.

XLMTM, X-linked myotubular myopathy; HCP, healthcare provider.

Supplementary Table 4 EQ-5D-5L index scores and VAS of participants

| **Participant number** | **1** | **4** | **5** | **6** | **8** | **13** | **14** | **15** | **16** | **17** | **21** | **22** |
| --- | --- | --- | --- | --- | --- | --- | --- | --- | --- | --- | --- | --- |
| Caregiver age | 28 | 44 | 34 | 43 | 42 | 45 | 50 | 47 | 49 | 39 | 50 | 62 |
| Sex | F | M | F | F | F | M | F | F | M | F | F | M |
| Caregiving years | <1 | 3* | 4 | 6 | 7 | 18 | 18 | 18 | 22 | 23 | >25 | >25 |
| Q1 – Mobility | 1 | 1 | 1 | 1 | 1 | 1 | 5 | 5 | 1 | 1 | 3 | 3 |
| Q2 – Self-care | 1 | 1 | 1 | 1 | 1 | 1 | 5 | 5 | 1 | 1 | 2 | 3 |
| Q3 – Usual activity | 4 | 4 | 2 | 4 | 2 | 1 | 4 | 5 | 1 | 1 | 2 | 3 |
| Q4 – Pain/discomfort | 1 | 1 | 1 | 1 | 3 | 2 | 3 | 1 | 2 | 2 | 3 | 3 |
| Q5 – Anxiety/depression | 3 | 1 | 2 | 2 | 4 | 2 | 4 | 2 | 1 | 2 | 1 | 3 |
| VAS | 83 | 85 | 85 | 80 | 58 | 78 | 80 | 60 | 88 | 80 | 38 | 47 |
| EQ-5D-5L index value (0–1) | 0.759 | 0.764 | 0.864 | 0.764 | 0.644 | 0.820 | 0.096 | 0.165 | 0.861 | 0.820 | 0.714 | 0.597 |
| US general population utility age/sex | 0.916 | 0.841 | 0.916 | 0.845 | 0.845 | 0.825 | 0.807 | 0.807 | 0.825 | 0.845 | 0.807 | 0.807 |
| **Disutility** | **–0.157** | **–0.0770** | **–0.052** | **–0.081** | **–0.201** | **–0.005** | **–0.711** | **–0.642** | **0** | **–0.025** | **–0.093** | **–0.21** |

*Died <2 years ago.

Non-negative values indicate no disutility and are set to 0.

Abbreviations: EQ-5D-5L, EuroQol 5-dimension 5-level; F, female; M, male; VAS, visual analog scale; XLMTM, X-linked myotubular myopathy.
